# The role of residence time in diagnostic models of global carbon storage capacity: model decomposition based on a traceable scheme

**DOI:** 10.1038/srep16155

**Published:** 2015-11-06

**Authors:** Chen Yizhao, Xia Jianyang, Sun Zhengguo, Li Jianlong, Luo Yiqi, Gang Chengcheng, Wang Zhaoqi

**Affiliations:** 1 School of Life Science, Nanjing University, Nanjing, P.R. China; 2 Department of Microbiology and Plant Biology, University of Oklahoma, OK, USA; 3 School of Ecological and Environmental Sciences, East China Normal University, Shanghai, P.R. China; 4 Tiantong National Forest Ecosystem Observation and Research Station,School of Ecological and Environmental Sciences, East China Normal University, Shanghai, P.R. China; 5 College of Prataculture Science, Nanjing Agriculture University, Nanjing, P.R. China; 6 Institute of Soil and Water Conservation, Northwest A&F University, Yangling, Shaanxi, China; 7 Institute of Soil and Water Conservation, Chinese Academy of Science and Ministry of Water Resources, Yangling, Shaanxi, China

## Abstract

As a key factor that determines carbon storage capacity, residence time (τ_E_) is not well constrained in terrestrial biosphere models. This factor is recognized as an important source of model uncertainty. In this study, to understand how τ_E_ influences terrestrial carbon storage prediction in diagnostic models, we introduced a model decomposition scheme in the Boreal Ecosystem Productivity Simulator (BEPS) and then compared it with a prognostic model. The result showed that τ_E_ ranged from 32.7 to 158.2 years. The baseline residence time (τ′_E_) was stable for each biome, ranging from 12 to 53.7 years for forest biomes and 4.2 to 5.3 years for non-forest biomes. The spatiotemporal variations in τ_E_ were mainly determined by the environmental scalar (ξ). By comparing models, we found that the BEPS uses a more detailed pool construction but rougher parameterization for carbon allocation and decomposition. With respect to ξ comparison, the global difference in the temperature scalar (ξ_t_) averaged 0.045, whereas the moisture scalar (ξ_w_) had a much larger variation, with an average of 0.312. We propose that further evaluations and improvements in τ′_E_ and ξ_w_ predictions are essential to reduce the uncertainties in predicting carbon storage by the BEPS and similar diagnostic models.

Terrestrial biosphere models (TBMs) are very important instruments for studying global and regional ecosystem variations and responses to climatic change^1^. In the past few decades, TBMs have undergone a rapid development. These models evolved from simple conceptual/statistical models into multi-disciplinary combined process-based models. For example, for carbon inflow calculation, early TBMs such as the Miami model^2^ used a simple relationship between photosynthesis and meteorological factors (i.e. air temperature and precipitation) to predict vegetation production. With the development of spatial technology and biogeochemical modeling, process-based photosynthesis and canopy scheme have been incorporated since the late 1980s^3^,^4^,^5^. Thereafter, model parameterization was further refined by combining the continuous measurements of flux sites and satellite remote sensing data^6^,^7^,^8^. With the amalgamation of plant geography, land surface biophysics and biogeochemical models, numerous aspects of terrestrial processes related to the terrestrial carbon cycle can be considered and simulated^9^. According to the latest incomplete statistics from Fisher, *et al.*^10^, there are now more than eighty TBMs.

With the increasing number of biogeochemical processes and corresponding parameters incorporated into TBMs, the model set is much closer to the real world processes. However, results vary greatly among different models at global or regional scales. A comparison of 16 TBMs showed that the global mean simulated net primary productivity (NPP) ranged from 39.9 Pg C yr^−1^ to 80.5 Pg C yr^−1^. In addition, Keenan, *et al.*^11^ compared the results of 17 models against flux sites in North America and showed that none of these models reliably reproduced the temporal variability in terrestrial carbon storage. A comparison of 7 diagnostic models showed that most models under-estimated carbon inflow^12^. Because the models are very complex, it is difficult to directly analyze which aspect of the models or sub-models mainly account for the discrepancy and to identify exactly where we should aim to improve model simulation.

Terrestrial carbon storage capacity is determined by carbon flux and carbon residence time^13^. NPP has been extensively studied in the past few decades, whereas studies on residence time, i.e., the time span that a carbon atom remains in a reservoir, are limited. This is recognized as an important source of uncertainty in model simulation. A recent analysis of 7 TBMs showed that limited understanding in residence time estimations cause the major uncertainties. residence time estimations dominate the uncertainties in simulating future terrestrial response to climatic change^14^. For Coupled Model Inter-comparison Project Phase 5 (CMIP5) models, the mean residence time result was under-estimated by 36% compared with observation-based estimates and the model could not reproduce the strong correlation between residence time and precipitation^15^. Several methods have been developed to estimate residence time, such as carbon isotope tracing technology^16^, inverse analysis^17^,^18^ and the calculation of the ratio of carbon storage in a specific pool to the corresponding carbon flux^15^,^19^. However, none of these methods are currently fully incorporated into TBMs for global simulations.

Basically, residence time includes all of the carbon allocation, transition and decomposition processes between different carbon pools. The decomposition and allocation of litter pools are determined by the physical and chemical properties of the plant detritus (lignin content and the ratio of carbon to nitrogen). Soil texture is important in the decomposition rate in soil carbon pools because this variable determines the physical and biochemical protection properties^20^. Together, these factors defined the potential decomposition rate (the inverse of baseline residence time) in an ecosystem. In addition, the potential decomposition rates of each carbon pool are modified by environmental scalars (e.g., temperature and moisture supply). Thus, the carbon storage ability (*C*_*st*_) can be represented using the following function:


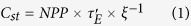


where NPP represents the carbon inflow, τ′_E_ represents the baseline residence time and ξ represents the environmental scalar.

Xia, *et al.*^21^ developed a traceable scheme for TBMs, which decomposed TBMs into independent components in an analytical way. Specifically, the terrestrial carbon storage ability is separated into carbon inflow and residence time, and then the latter component is further separated into **τ**′_**E**_ and **ξ** of the decomposition. The baseline residence time is determined by carbon allocation in the vegetation pools (Vector B), a transfer matrix between different pools (Matrix A) and the maximum decomposition rates in different carbon pools (Matrix C), whereas the environmental scalar is the combined effect of soil temperature and moisture factors on the decomposition rate.

Diagnostic models and prognostic models are two important groups of TBMs. Although they follow similar general model concepts to describe ecosystem processes, differences exist in both the NPP and residence time simulation. Diagnostic models use historical satellite data as a model input to monitor spatiotemporal carbon inflow. The use of measured data could constrain canopy dynamics and thus largely enhance the prediction of carbon influx. A general example is the MODIS NPP product, which has been used as an important criterion for many model evaluations and validations^22^,^23^. However, because the canopy condition is related to many ecological processes, the incorporation of external data requirements could, at the same time, influence the simulation of residence time. For instance, leaf biomass is largely pre-defined by data input, hence the leaf pool should not be considered in NPP allocation simulations; plant phenology is implicitly included by remote sensing inputs and consequently, the external influences on plant decomposition are difficult to identify. Prognostic models, however, have its nature merit to simulate these processes without concern for the preset canopy condition. Thus, by comparing the corresponding decomposition components with a prognostic model, it could be much easier to identify the major differences and determine what is needed to improve the performance of residence time simulation in diagnostic models.

In this study, we initially introduced the traceable scheme into the BEPS to assess how residence time was simulated in the current version. Specifically, the model codes were reorganized into matrix functions to represent vegetation carbon allocation, carbon transition, maximum carbon decomposition rates and environmental scalars in different carbon pools. Thereafter, we compared the results with those of the Community Atmosphere Biosphere Land Exchange model (CABLE, a prognostic model) to analyse the potential sources of uncertainty in residence time simulation in diagnostic models.

## Results

### Traceable components in the BEPS

#### Terrestrial carbon storage capacity regulated by NPP and residence time

**Figure 1:**
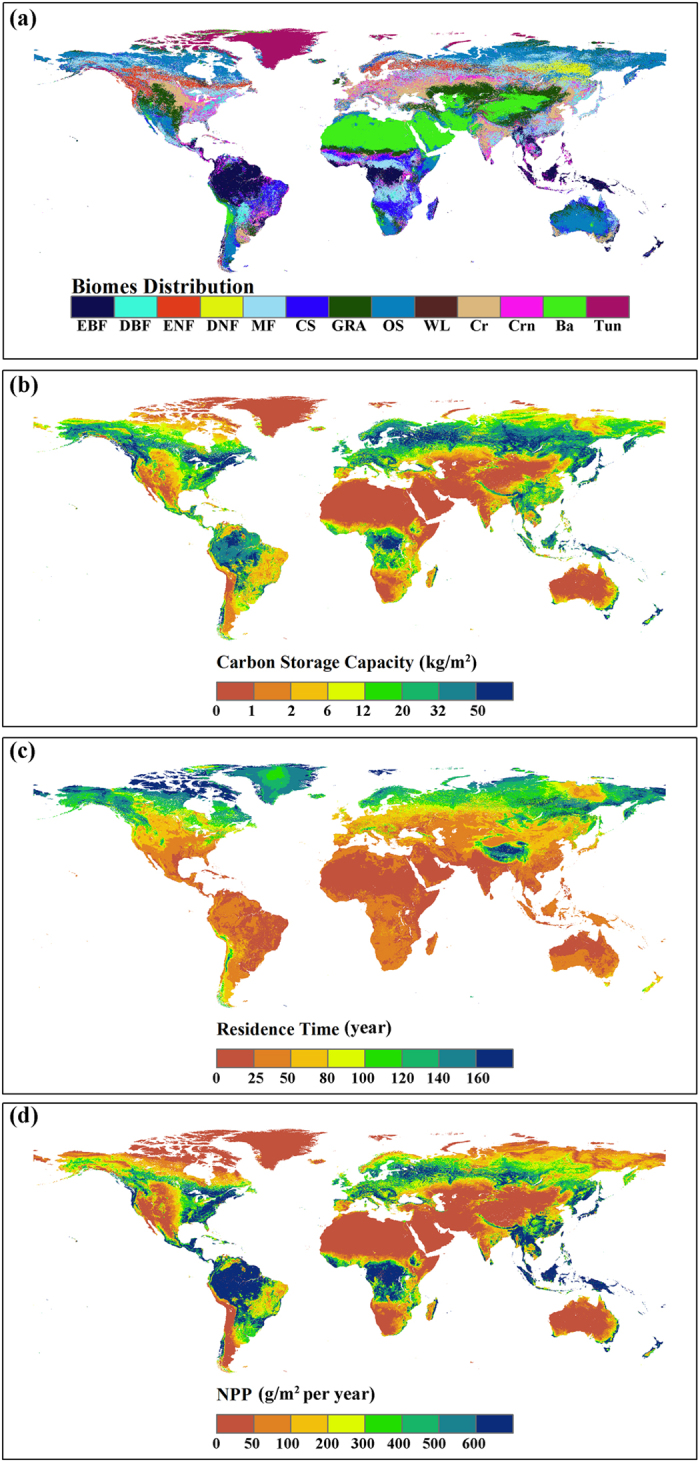
Global distribution of (**a**) biomes, (**b**) carbon storage capacity, (**c**) residence time and (**d**) NPP. EBF: evergreen broadleaf forest, DBF: deciduous broadleaf forest, ENF: evergreen needleleaf forest, DNF: deciduous needleleaf forest; MF: mixture forest; CS: close shrub; GRA: grassland; OS: open shrub; WL: wetland; Cr: crop; Crn: crop/nature; Ba: barren; Tun: tundra (this figure was created from authors’ data with ArcGis 10.0 software (ESRI): www.esri.com/software/arcgis/).

**Figure 2:**
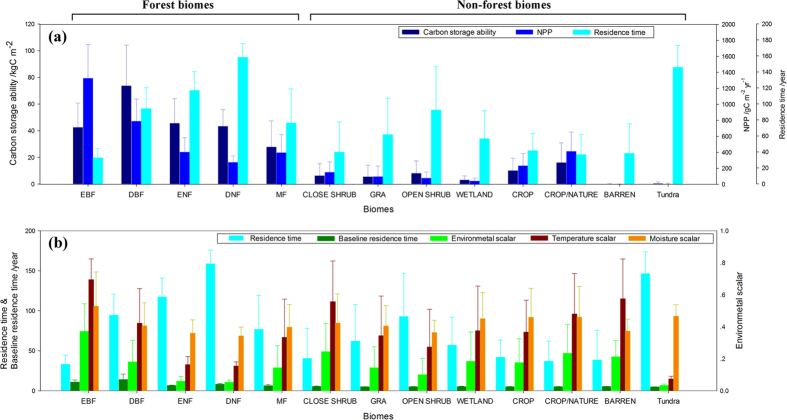
Traceable components of global terrestrial carbon storage in the various biomes based on the BEPS. (**a**) Mean carbon storage capacity, residence time and NPP. (**b**) Mean value and variation range of residence time and baseline residence time. (**c**) Mean environmental scalar, temperature scalar and moisture scalar. EBF: evergreen broadleaf forest, DBF: deciduous broadleaf forest, ENF: evergreen needleleaf forest, DNF: deciduous needleleaf forest; MF: mixture forest; GRA: grassland.

Globally, the overall mean NPP is 272.9 gC m^−2^ yr^−1^ and the mean residence time is 69.1 years, which results in a mean carbon storage ability of 18.9 kgC m^−2^. Terrestrial carbon is stored mainly in forest biomes with 72.8% (Figs 1 and 2a). Among the forest biomes, evergreen broadleaf forest (EBF) exhibited the highest NPP, i.e., 1325.7 gC m^−2^ yr^−1^. This forest biome is mainly located in tropical rain forest areas (Amazonian and African rain forests) and thus takes advantage of the high average temperature and abundant rainfall (Fig. 1a and Supplementary Table S1). However, its residence time is the lowest in the forest ecosystem (32.7 years), which results in an intermediate total carbon storage capacity among the forest biomes (42.5 kgC m^−2^). Deciduous needleleaf forest (DNF) is mainly located in central of Siberia under relatively harsh environmental conditions; therefore, its mean NPP is only about one quarter of the EBF value (275.8 gC m^−2^ yr^−1^) (Figs 1d and 2a). However, DNF has the highest residence time (158.2 years) of all of the biomes, which makes its carbon storage capacity very similar to that of EBF (43.3 kgC m^−2^) (Fig. 1c). Deciduous broadleaf forest (DBF) had the highest carbon storage capacity; this forest biome is scattered across the temperate areas of Southeast North America, East Asia and Central South America (Fig. 1b). The temperature in these regions is intermediate, and the rainfall is sufficient (see Supplementary Table S1). DBF had a relatively high NPP (789.3 gC m^−2^) and residence time (94 years).

Crop ecosystems (including cropland and cropland/natural mosaics) exhibit high carbon inflows (231.3 gC m^−2^ yr^−1^ and 412.5 gC m^−2^ yr^−1^, respectively) in non-forest biomes. However, their residence times are low (41.4 years and 36.6 years, respectively), which corresponds to relatively low carbon storage abilities (10.1 kgC m^−2^ and 16.1 kgC m^−2^, respectively) (Fig. 2a). The Tundra biome (Tundra) has a very high residence time of 145.9 years, but its carbon flux is very low (2.5 gC m^−2^ yr^−1^), which makes it difficult to store carbon. Both the residence time and NPP are very low in the Barren biome (Barren), resulting in the lowest carbon storage (44.9 gC m^−2^).

#### Residence time regulated by baseline residence time and environmental scalars

The residence time is determined by the baseline residence time and environmental scalars (Equation 5). The residence times range from 32.65 years (EBF) to 158.22 years (DNF) (Fig. 2b). In forest biomes, the high residence time in DNF originates from the high baseline residence time (53.7 years) and intermediate environmental scalar value (0.18). On the contrary, the low residence time in EBF originates from the low baseline residence time (18.7 years) and high environmental scalar value (0.37). The baseline residence times are very similar among non-forest biomes. Therefore, the high residence times in the Tundra and Open shrub biomes (145.9 and 92.3 years, respectively) are due to their very low environmental scalar values (0.03 and 0.1, respectively). The remaining biomes showed similar residence times, ranging from 36.6 (Crop) to 61.6 (Grassland) years.

According to Fig. 2b, we found that the baseline residence time is very stable for each biome. Both the mean value and the standard deviation of the residence time are much larger than those of the baseline residence time, especially for non-forest biomes. Therefore, in non-forest biomes, the differences in the residence times can be attributed mainly to the environmental scalar. Meanwhile, the large spatial variability of the residence time should also be attributed to the wide ranges of the environmental scalar.

#### Baseline residence time

The baseline residence time was calculated by using the potential decomposition rate (*C* matrix), the transfer rates between different pools (*A* matrix) and carbon allocation to vegetation pools (*B* vector) (Equation 4). In the model, the *C* and *A* matrices were partly modified using the lignin to nitrogen ratio (L/N), the lignin content in different plant components (i.e., Lleaf, Lfroot and Lwood) and the soil texture (i.e., clay and silt contents). In the current BEPS version, the L/N ratio and lignin content were predefined; therefore, the spatial variability of the intra-biomes is attributed to the model input of soil texture.

According to the *C* matrix, the potential turnover rate is very low for woody and coarse root pools, which means that the carbon from these two pools would decompose much slower than that from the leaves and fine roots (See description in Supplementary information). In the litter pools, the potential turnover rate of the coarse detritus pool (carbon mainly transferred from woody and coarse root pools) is also the lowest.

The baseline residence time ranges from 4.18 years (Grassland) to 53.7 years (DBF) (Fig. 2b). The values are approximately 4–6 years for non-forest biomes and from 12 to 53.7 years for forest biomes. The higher value for forests is due to the greater allocation of carbon to the woody compartment (0.3–0.5).

A sensitivity analysis showed that the baseline residence time is sensitive to the L/N ratio, the lignin contents (Lleaf, Lfroot, Lwood) and the soil texture in all biomes (see Supplementary Table S2). The highest sensitivity (S value) was observed with respect to the lignin contents, followed by the L/N ratio and the soil texture.

#### Environmental scalar

The environmental scalar ranges from 0.034 (Tundra) to 0.37 (EBF), where the temperature scalar ranges from 0.073 (Tundra) to 0.69 (EBF) and the moisture scalar ranges from 0.34 (DNF) to 0.53 (EBF) (Fig. 2c).

The environmental scalars showed diverse results for different forest biomes. evergreen needleleaf forest (ENF) and DNF are mainly located at the high latitude areas in the Northern Hemisphere (Fig. 1a). Their temperature scalar is among the lowest across all biomes due to the low temperature condition (see Supplementary Table S1). EBF has the highest environmental scalar value, with both the highest temperature and moisture scalars (Fig. 2b). For the non-forest biomes, the Tundra and Open shrub biomes exhibited very low values due to their locations close to the Polar Regions. The environmental scalars for the other biomes are moderate, ranging from 0.14 to 0.24.

### Comparison of residence time components between the BEPS and the CABLE model

Both models use the same model concept to calculate the residence time. The CABLE model uses a simpler scheme consisting of 9 pools (3 vegetation pools and 6 litter and soil pools, Kowalczyk, *et al.*^24^), while BEPS uses a 13-pool scheme. For the *A* and *C* matrices in the baseline residence time simulation, the L/N ratio determines the transfer strategy of carbon from the vegetation pools to the litter pools; the lignin content in different vegetation components modifies the potential decomposition rates and transfer strategy between the litter and soil pools; the soil texture regulates the carbon allocation of the soil pools. In the BEPS, the L/N ratio and lignin contents are predefined values for all biomes, whereas the spatial variations are considered in the CABLE model. The seasonal variation of the leaf decomposition rate is not considered in the current BEPS. For the *B* matrix, the BEPS uses preset values for different biomes, whereas the CABLE model uses a multi-limitation partitioning scheme to simulate carbon allocation (Table 1).

**Figure 3:**
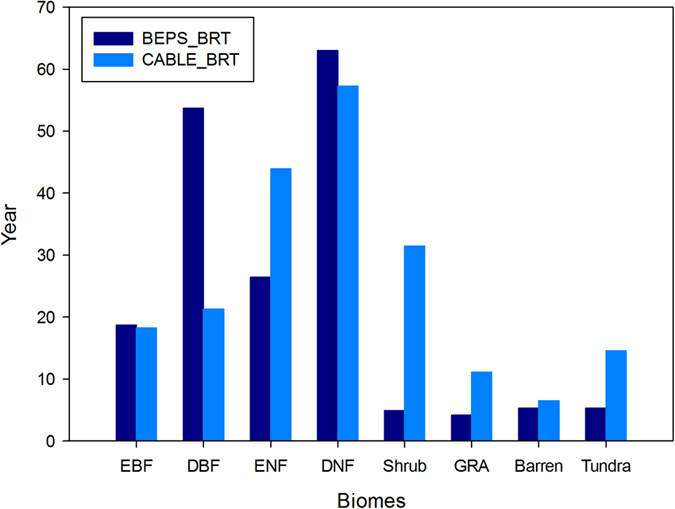
Comparison of the baseline residence times from the BEPS and CABLE models for the same biomes considered in both studies. BEPS_BRT, CABLE_BRT: baseline residence time from the BEPS and CABLE models, respectively.

Using these different model sets, the baseline residence time exhibited large discrepancies between the two models (Fig. 3). For the forest biomes, the baseline residence time of DBF in the BEPS is obviously higher than that in the CABLE model (53.7 versus 21.3 years), while the ENF value is much higher in the CABLE model than in the BEPS (43.9 versus 26.4 years). For EBF and DNF, the BEPS results are slightly higher than those of the CABLE model (Fig. 3). For the non-forest biomes, the BEPS are very similar, ranging from 4.18 years in Grassland (GRA) to 5.3 years in the Tundra, while in the CABLE model, the values range from 6.47 years in the Barren regions to more than 30 years in the Shrub regions.

**Figure 4:**
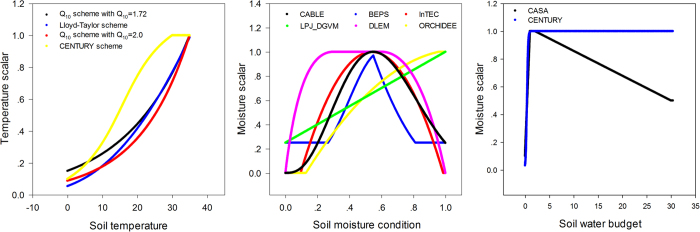
Temperature and moisture scalar variations with soil temperature and the moisture condition in the BEPS and CABLE models, respectively. Please notice that different models use different variables to represent the moisture condition and water budget (See detailed descriptions in Table 2).

The two models use different schemes for the temperature and moisture scalars. For the temperature scalar, the BEPS uses the Lloyd-Taylor algorithm (i.e., 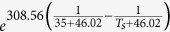
25), while the CABLE model uses the standard Q_10_ algorithm (i.e., 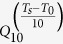
3). The two algorithms are widely applied in many TBMs^26^. We found that for these two algorithms, the temperature scalar shows a similar pattern with the same soil temperature input, and the discrepancy decreases with increasing soil temperature (Fig. 4). For the moisture scalar, the CABLE model uses the soil water content as the fraction for the saturated condition, and one function is used for the entire range. The BEPS uses the ratio of soil water content and soil porosity (calculated from soil texture) as the independent variable as well as a subsection model with a predefined lower limit set equal to 0.25. In addition, factors for dry and cold conditions are considered for leaf decomposition in the CABLE model^24^.

**Figure 5:**
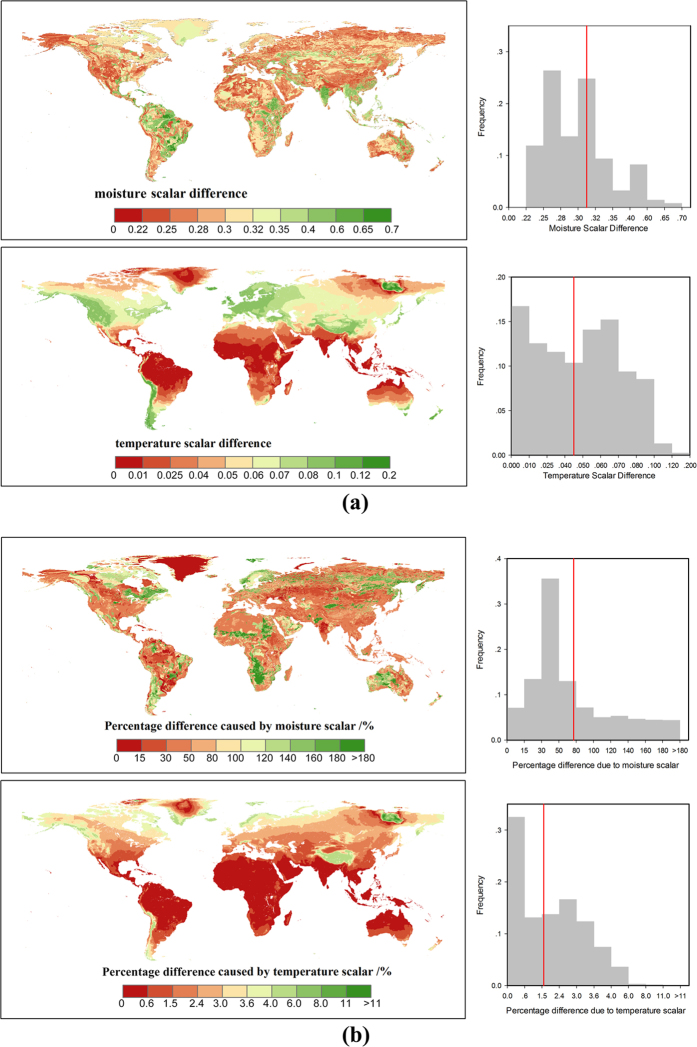
Global spatial and frequency distributions of (**a**) temperature scalar and moisture scalar differences between the BEPS and CABLE models and (**b**) carbon storage and residence time differences caused by using different environmental scalars in the BEPS and CABLE models (The global spatial distribution component was created using ArcGis 10.0 software (ESRI): www.esri.com/software/arcgis/).

By using the CABLE model temperature scheme in the BEPS, the global mean difference is 0.045 compared with the original BEPS scheme (Fig. 5a). In biomes view, the influence ranges from 0.013 in EBF to 0.074 in ENF (see Supplementary Fig. S1). However, for the moisture scalar, the results showed much larger discrepancy when the CABLE model scheme was applied; the mean global difference is 0.312, ranging from 0.288 in Barren to 0.382 in EBF (see Supplementary Fig. S1). Based on this result, a change in the temperature scalar changes the global carbon storage ability and residence time by 1.67%. The corresponding change reaches 72.6% with a different moisture scalar (Fig. 5b). The carbon storage ability of more than 25% of terrestrial areas was changed by more than 100%. These areas are mainly distributed in the barren regions in Central Africa, Central Asia and Central Australia, and the forests in South Africa, Eastern North America and Eastern Siberia with high precipitation and carbon inflow.

## Discussion

### Traceability of carbon storage ability in diagnostic models

Decompose the model into independent components facilitates understanding of the spatial distribution of terrestrial carbon storage and how residence time regulates carbon storage ability across biomes. In the BEPS, because the baseline residence time is mainly calculated from preset values, the residence time is largely regulated by environmental scalars, especially in non-forest biomes. For example, we found that ENF, which is mainly located in high latitude areas of the North Hemisphere, showed a very similar carbon storage capacity to EBF, which is mainly located in the Amazon region and Central Africa. The NPP of ENF is less than one third that of EBF (402.4 gC m^−2^ versus 1325.7 gC m^−2^). However, the temperature scalar of ENF is low because of the cold conditions, which results in a considerably smaller environmental scalar than that of EBF (0.05 versus 0.37). Therefore, although their baseline residence times are not markedly different (18.7 versus 26.4 years), the ENF residence time is much higher than that of EBF (116.8 versus 32.7 years). Due to this model feature, on one hand, accurate simulation of the environmental scalar is important for the residence time results in the current version of the model; on the other hand, the residence time and its response to climatic change is relatively simple and largely predictable. For example, the residence time is positively correlated with the soil temperature and low soil moisture but negatively correlated with high soil moisture (Fig. 4). Other effects like CO_2_ and N deposition are not considered.

One of the distinctive features of a diagnostic model, as mentioned in the introduction section, is the input from remote sensing datasets, such as canopy information. For example, the BEPS uses remote sensing leaf area index (LAI) for the scaling transformation of carbon inflow from leaf level to ecosystem level, and the CASA (i.e., Carnegie-Ames-Stanford approach^5^) uses Normalized Difference Vegetation Index (NDVI) or Enhanced Vegetation Index (EVI) to calculate fraction of Photosynthetic Active Radiation (fPAR)^5^,^27^. Using this remote sensing input, the processes of vegetation phenology (e.g., plant senescence and mortality) do not need to be simulated, which in turn, greatly reduces the uncertainty of the carbon influx calculation^28^. Moreover, with the help of remote sensing inputs, the models implicitly include some major disturbance effects on vegetation (e.g., fire, deforestation and grazing activity), which could further reduce the uncertainty related to ignorance or misrepresentation of these unconventional events in large scale simulations^29^,^30^. However, the inclusion of multi-processes using remote sensing input can result in certain limitations in the residence time simulation. Because the canopy condition is pre-fixed, some processes are not efficiently simulated. A comparison with the prognostic model indicated that carbon allocation (*B* vector), the environmental limitations on decomposition rates and the seasonal variation of potential decomposition of vegetation pools (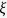 matrix and *C* matrix) were not well represented using process-based functions. As a result, the baseline residence time could not be well simulated. An obvious evidence is the similarity of the baseline residence time in all non-forest ecosystems worldwide, which is very different from the experimental results^31^. These limitations may also exist widely in similar diagnostic models like CASA-AMES, GLOPEM-CEVSA (i.e., Global Production Efficiency Model with the Carbon Exchange in the Vegetation-Soil-Atmosphere^32^) and inTEC (i.e., Integrated Terrestrial Ecosystem C-budget model^33^). Specifically for BEPS, other improvements may also need to be implemented, including the specification of sensitive parameters in the baseline residence time simulation (e.g., the L/N ratio and lignin content in different vegetation components). In addition, model coupling of carbon related nutrient (e.g., nitrogen and phosphorus) cycles should also be considered in future model development.

### Large uncertainty exists in the moisture scalar simulation

According to the review conducted by Fisher, *et al.*^10^, most TBMs use temperature and moisture scalars to calculate the environmental scalar, but the model schemes of the two scalars vary and exhibit different trends. With the same forcing data and soil temperature simulation scheme in the BEPS, the two temperature scalar algorithms of the BEPS and CABLE model showed relatively similar global patterns. However, for the moisture scalar, the results showed a large discrepancy between the two schemes with respect to the global simulation. This means that the difference in the moisture scalar scheme could largely contribute to the final discrepancy of the environmental scalar, residence time and carbon storage capacity. Following this result, we further checked the environmental scalar algorithms in certain widely used TBMs (Table 2, Fig. 4). The results show that in current TBMs, the temperature scalar algorithms are very similar and has been widely studied^34^,^35^. The models mainly use the Lloyd-Taylor scheme or Q_10_ scheme (the only exception is the CENTURY model). However, for the moisture scalar calculation, the algorithms are quite different. First, different variables are used to represent the soil moisture condition in the various models. For example, in the IBIS (i.e., the Integrated Biosphere Simulator^36^), ORCHIDEE (i.e., Organizing Carbon and Hydrology in Dynamic Ecosystems^37^) and DLEM (i.e., Dynamic Land Ecosystem Model^38^), the soil water content is used directly, whereas in BEPS and inTEC, the soil porosity is considered to represent the available soil water content, while in the CASA-AMES and CENTURY/daycent models, the soil moisture budget (the ratio between the water input and output) is used to calculate the scalar. Second, the schemes and algorithms to calculate the moisture scalar vary. Some models use two or three sections with different equations, while other models only use one equation. We found certain differences by plotting the relationship between the soil moisture condition and the moisture scalar, In the ORCHIDEE, LPJ-DGVM (i.e., Lund-Potsdam-Jena Dynamic Global Vegetation Model^39^) and CENTURY, the scalar generally increased linearly with the soil moisture condition. These models may cause uncertainty because both drought and over-hydration conditions could largely inhibit microbial activities^40^,^41^. Although other models consider these effects by using parabolic-type models, their discrepancies are very large for extreme conditions (Fig. 4), which were also identified through our model comparison (Fig. 5). By testing the IBIS (same function with different moisture variable) and ORCHIDEE scheme (linear function) in the BEPS, we found that different use of moisture variables and functions will cause certain differences on the model outputs (see Supplementary Fig. S2–4). According to our knowledge, no standard has been set to evaluate the accuracy of the simulated moisture scalar. Hence, it could be an important source of uncertainty that generally exists in both single model simulation and inter-comparison. It is critical than an evaluation be conducted for the BEPS because its residence time is largely controlled by the environmental scalar.

### Future perspective on the traceable scheme and model benchmarking

Because many historical model revisions and improvements have concentrated on the simulation of NPP, it is necessary to decompose the carbon inflow into several independent components. For diagnostic models, we propose that the next step is to split the remote sensing inputs and the instant photosynthesis simulation. However, NPP cannot be decomposed directly because autotrophic respiration (R_a_) has to be subtracted from gross primary productivity (GPP). A possible solution is to rearrange R_a_ into the *A* matrix (consider R_a_ as a transfer factor) and modify the input of the transfer rates from the vegetation to the litter pools.

Technically, our model comparison set in this study is not comprehensive (We compared the model scheme of residence time in only two models). By incorporating more models with the same forcing data in the future, it will be possible to use the traceable scheme to determine where the major discrepancy exists among different diagnostic models, or even TBMs. In this way, we could specifically identify where we need to focus on to improve the model performance on terrestrial ecosystem simulations.

Finally, to contribute to single model improvement or multi-model benchmarking, datasets based on observations are necessary. Currently, more and more components in ecosystem processes and properties could be evaluated by well-organized observations^1^,^43^. However, although the issue of carbon residence time simulation has been recognized for a certain period of time, real considerations and systematic evaluations on regional or global scales are still new for the international research community. Observations of residence time and its sub-components are very limited, and no benchmarking database has been produced for model validation and evaluation. Therefore, we anticipate that this study will encourage future studies on the mechanical understanding of residence time and thus contribute to the potential development of benchmarking datasets for this model component.

## Materials and Methods

### Model description

The BEPS, developed by Liu, *et al.*^44^ and Chen, *et al.*^45^, was the first model to be developed to simulate boreal ecosystem productivity; a CENTURY model-derived soil scheme was incorporated into the model to account for R_h_ and NEP^45^, and a terrestrial hydrological and energy model was included to account for the soil water content (SWC) and evapotranspiration^47^,^48^.

The model has 13 pools, including 4 vegetation pools (woody, leaf, fine root, and coarse root), 5 litter pools (surface metabolism, surface structure, soil metabolism, soil structure, and coarse detritus), and 4 soil pools (surface microbe, soil microbe, slow, and passive). Each pool has a pre-defined turnover rate (the reciprocal of the residence time). In this study, we used a version of the BEPS that is coupled with the soil carbon, water and energy modules. This version has been applied and validated in numerous global and regional studies and in recent comparisons^49^,^50^,^51^.

### Introduction of the traceable scheme and its components to the BEPS

The model traceable scheme was first developed by Xia, *et al.*^21^. Basically it follows the conceptual model of Luo, *et al.*^17^:


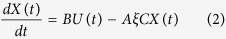


The left side of the equation represents the change in each carbon pools in per unit time, *t*. In the BEPS, there are 13 pools; therefore, *X(t)* is a 13 × 1 vector that represents one pool size at a time. *BU*(t) represents the carbon influx into the system and its allocation per unit time, and *U(t)* is the net carbon influx, generally NPP. *B* = (b1, b2, b3, b4, 0, 0,.., 0)^*T*^ is a 13 × 1 vector that represents the NPP allocation to different vegetation components.


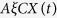
 represents the carbon outflow from the system. Matrix *A* is the carbon transfer matrix among different pools:


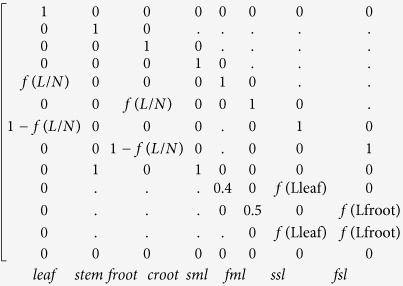



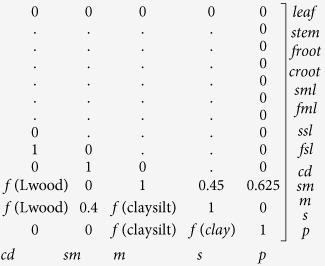


In matrix *A*, the leaf and fine root pools are decomposed into the surface and soil metabolic pools. The rate of carbon allocation is determined by the ratio of the initial residue lignin to the nitrogen content (L/N), which is preset in the BEPS. The stem and coarse root pools are transferred into the coarse detritus pool. The transformation rates among the litter and soil pools are functions of the soil texture (*f*(clay_silt), *f*(clay)) and the lignin contents in the different vegetation components (*f*(Lleaf), *f*(Lfroot), *f*(Lwood))

Matrix ξ is a 13 × 13 diagonal matrix with diagonal input by vector ξ’ = (1, 1, 1, 1, ξ’_5_, …ξ’_13_), which quantifies the environmental scalar for the carbon decomposition of each pool. Matrix *C* is a 13 × 13 diagonal matrix with diagonal input by vector *c* = (c1, c2, c3…, c13)^*T*^, which defines the potential turnover rate for each pool. A detailed description of each component can be found in Text S1 in Supplementary information.

Therefore, based on this model scheme, each model component at steady state (i.e., 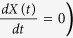 can be calculated as:


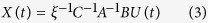


*X(t)* can be calculated as the combination of *U(t)* and the residence time (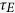); therefore, 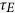
can be calculated as:


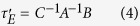


The residence time (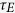) is calculated from the baseline residence time (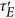) and environmental scalar (ξ):


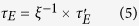


ξ can be further decomposed into the soil moisture scalar (ξ_w_) and temperature scalar (ξ_t_):


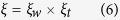


Finally, ξ_w_ and ξ_t_ are calculated from the model inputs of precipitation and temperature. In the BEPS, the limitation on vegetation pools is not considered, whereas the temperature and water limitations are considered in the litter and soil pools by using the same strategy.

### Model simulation and comparison

First, the model codes were rewritten to fit for the traceable scheme outputs. Thereafter, we ran the model to steady state using the forcing data from 1982 to 1986. The vegetation cover was based on the IGBP classification^52^. The daily meteorological dataset used the Global Meteorological Forcing Dataset for Land Surface Modeling archive (http://rda.ucar.edu/datasets/ds314.0/, 1982–1986). The GlobalLAI dataset (http://www.globalmapping.org/globalLAI/, 1982–1986) was used for the global LAI input. The soil textural data were obtained from the Global Soil Dataset for use in Earth System Models (GSDE, available at: http://globalchange.bnu.edu.cn/research/soilw). All of these input data were resampled to 8 km×8 km spatial resolution.

The BEPS uses a spin-up scheme that is very similar to the semi-analytical scheme (SAS) developed by Xia, *et al.*^53^. That is, instead of running the model for thousands of years, the model calculates the stable state based on the equilibrium hypothesis. Because the BEPS uses the remote sensing LAI values as model inputs, the vegetation pools do not need to be spun-up.

We compared the model schemes of residence time in the BEPS and a prognostic model, the Community Atmosphere Biosphere Land Exchange (CABLE) model. The baseline residence time results of the CABLE model were from Xia, *et al.*^21^ using the same land cover map. We only compared the natural biomes with available data from Xia’s work. To quantify the contribution of the model schemes of the environmental scalar to the final difference, we consecutively replaced the BEPS algorithms for the temperature and moisture scalars by the ones from the CABLE model, and then ran the model.

### Sensitivity analysis

To quantify the sensitivity of a number of parameters in the baseline residence time calculation, we applied a method from Williams, *et al.*^54^:


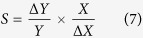


In this study, we evaluated the main inputs for the baseline residence time calculation including the ratio of the lignin to nitrogen in the litter (L/N) and the fraction of the lignin in the leaf (Lleaf), fine root (Lfroot) and woody (Lwood) parts. A specific parameter was considered sensitive to the model output if the |S| value exceeded 0.2.

## Additional Information

**How to cite this article**: Yizhao, C. *et al.* The role of residence time in diagnostic models of global carbon storage capacity: model decomposition based on a traceable scheme. *Sci. Rep.* **5**, 16155; doi: 10.1038/srep16155 (2015).

## Supplementary Material

Supplementary Information

## Figures and Tables

**Table 1:** Comparison of the model set of residence times between the BEPS and CABLE models.

Model Name		BEPS	CABLE
Pool Number		13	9
Baseline Residence Time	*A* matrix and *C* matrix	L/N, lignin content: unique values; Soil texture: data input	L/N: spatiotemporal variation; Lignin content: vary with vegetation types; Phenology: spatiotemporal variation; Soil texture: data input
	*B* vector	Varies with vegetation types	Varies with pixels (multi-limitation partition scheme)
Environmental Scalar		Litter and soil pools: Soil temperature and moisture scalar	Leaf pool: air temperature and water scalar; Litter and soil pools: Soil temperature and moisture scalar

**Table 2:** Moisture and Temperature scalar algorithms in different Terrestrial Biosphere Models.

Model name	Moisture scalar	Temperature scalar	References
BEPS	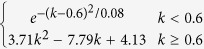	Lloyd-Taylor scheme	Ju, *et al.*^45^
CASA-AMES/NASA-CASA	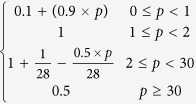	Q_10_ scheme	Potter, *et al.*^5^,^27^
inTEC	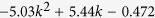	Lloyd-Taylor scheme	Ju, *et al.*^48^
IBIS	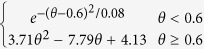	Lloyd-Taylor scheme	Foley, *et al.*^36^
LPJ-DGVM	0.25 + 0.75 W_1_	Lloyd-Taylor scheme	Sitch, *et al.*^39^
ORCHIDEE	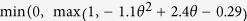	Q_10_ scheme	Krinner^37^
DLEM	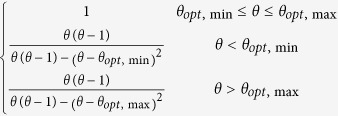	Lloyd-Taylor scheme	Tian, *et al.*^38^
CABLE	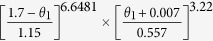	Q_10_ scheme	Kowalczyk, *et al.*^24^
CENTURY/daycent	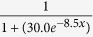	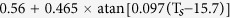	Parton, *et al.*^42^

θ in the IBIS, ORCHIDEE and DLEM models represents the soil water content; θ_1_ in the CABLE model represents the soil water content as a fraction of the saturated soil water content; k in the BEPS and inTEC models represents the SWC as a fraction of porosity; p in the CASA-AMES model represents (PPT + SOILW)/PET, PPT is precipitation, SOILW is current soil water storage, and PET is potential evaporation; W_1_ in the LPJ-DGVM model represents the SWC in the upper soil layer; x in the CENTURY/daycent model represents PPT/PET. The Q_10_ scheme represents the standard Q_10_ equation: 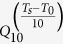, where Q_10_ is the soil respiration temperature sensitivity, T_s_ is the soil temperature, and T_0_ is the optimal soil temperature. The Lloyd-Taylor scheme represents the Lloyd-Taylor equation: 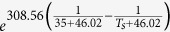.
