# Supplementary material for: The role of residence time in diagnostic models of global carbon storage capacity: model decomposition based on a traceable scheme
Source: Sci Rep. 2015 Nov 6;5:16155. doi: 10.1038/srep16155 (PMC4635433; doi:10.1038/srep16155)

Supplementary information for:

The role of residence time in diagnostic models of global carbon storage capacity: model decomposition based on a traceable scheme

Chen Yizhao[A], Xia Jianyang[B,C], Sun Zhengguo[A,D], Li Jianlong[A*], Luo Yiqi[B], Gang Chengcheng[A,E,F], Wang Zhaoqi[A]

[A] School of Life Science, Nanjing University, Nanjing, PR China

[B] Department of Microbiology and Plant Biology, University of Oklahoma, OK, USA

[C] School of Ecological and Environmental Sciences, East China Normal University, Shanghai, PR China

[D] College of Prataculture Science, Nanjing Agriculture University, Nanjing, PR China

[E] Institute of Soil and Water Conservation, Northwest A&F University, Yangling, Shaanxi, China

[F] Institute of Soil and Water Conservation, Chinese Academy of Science and Ministry of Water Resources, Yangling, Shaanxi, China

* Corresponding author.

Post address: School of Life Science, Nanjing University, Hankou Road 22, Nanjing 210093, PR China

Tel: +86-25-86214644; Fax: +86-25-83302728

Email address: jianlongli@gmail.com

Text S1. Description of components to calculate baseline residence time in BEPS.

Matrix A:

Matrix *A* is the carbon transfer matrix among different pools:

$$
\begin{array}{c}
\left(\begin{array}{ccccccccccccc}
1 & 0 & 0 & 0 & 0 & 0 & 0 & 0 & 0 & 0 & 0 & 0 & 0 \\
0 & 1 & 0 & . & . & . & . & . & . & . & . & . & 0 \\
0 & 0 & 1 & 0 & . & . & . & . & . & . & . & . & 0 \\
0 & 0 & 0 & 1 & 0 & . & . & . & . & . & . & . & 0 \\
f(L/N) & 0 & 0 & 0 & 1 & 0 & . & . & . & . & . & . & 0 \\
0 & 0 & f(L/N) & 0 & 0 & 1 & 0 & . & . & . & . & . & 0 \\
1-f(L/N) & 0 & 0 & 0 & . & 0 & 1 & 0 & . & . & . & . & 0 \\
0 & 0 & 1-f(L/N) & 0 & . & 0 & 0 & 1 & 0 & . & . & . & 0 \\
0 & 1 & 0 & 1 & 0 & 0 & 0 & 0 & 1 & 0 & . & . & 0 \\
0 & . & . & . & 0.4 & 0 & f(\text{Lleaf}) & 0 & 0 & 1 & 0 & . & 0 \\
0 & . & . & . & 0 & 0.5 & 0 & f(\text{Lfroot}) & f(\text{Lwood}) & 0 & 1 & 0.45 & 0.625 \\
0 & . & . & . & . & 0 & f(\text{Lleaf}) & f(\text{Lfroot}) & f(\text{Lwood}) & 0.4 & f(\text{clay\_silt}) & 1 & 0 \\
0 & 0 & 0 & 0 & 0 & 0 & 0 & 0 & 0 & 0 & f(\text{clay\_silt}) & f(\text{clay}) & 1
\end{array}\right)
\begin{array}{c}
leaf \\ stem \\ froot \\ croot \\ sml \\ fml \\ ssl \\ fsl \\ cd \\ sm \\ m \\ s \\ p
\end{array} \\
\begin{array}{ccccccccccccc}
leaf & stem & froot & croot & sml & fml & ssl & fsl & cd & sm & m & s & p
\end{array}
\end{array}
$$

In *A* matrix, leaf and fine root pools are decomposed into surface and soil metabolic litter pool (*sml* and *fml*, respectively). The rate of carbon allocation is determined by initial residue lignin to nitrogen content (L/N), which is preset in BEPS. Stem and coarse root pools (*stem* and *croot*, respectively) are all transfer into coarse detritus pool (*cd*). The transform rates among litter and soil pools are functions of lignin contents in different vegetation components (*f*(Lleaf), *f*(Lfroot), *f*(Lwood)) and soil texture (*f*(clay_silt), *f*(clay)) .

Vector B:

Vector B represents carbon allocation into leaf, stem, froot and croot pools. The partition coefficients are defined with different vegetation types (VT):

$$
\begin{pmatrix}
coef(\mathrm{VT}) \\
coef(\mathrm{VT}) \\
coef(\mathrm{VT}) \\
coef(\mathrm{VT}) \\
0 \\ 0 \\ 0 \\ 0 \\ 0 \\ 0 \\ 0 \\ 0 \\ 0
\end{pmatrix}
\begin{matrix}
leaf \\ stem \\ froot \\ croot \\ sml \\ fml \\ ssl \\ fsl \\ cd \\ sm \\ m \\ s \\ p
\end{matrix}
$$

Matrix C:

Matrix C represents the potential turnover rates in each pools:

$$
\begin{matrix} \\ \end{matrix}
\begin{pmatrix}
coef1(\mathrm{VT}) & 0 & 0 & 0 & 0 & 0 & 0 & 0 & 0 & 0 & 0 & 0 & 0 \\
0 & coef1(\mathrm{VT}) & 0 & . & . & . & . & . & . & . & . & . & 0 \\
0 & 0 & coef1(\mathrm{VT}) & 0 & . & . & . & . & . & . & . & . & 0 \\
0 & . & 0 & coef1(\mathrm{VT}) & 0 & . & . & . & . & . & . & . & 0 \\
0 & . & . & 0 & 14.8 & 0 & . & . & . & . & . & . & 0 \\
0 & . & . & . & 0 & 18.5 & 0 & . & . & . & . & . & 0 \\
0 & . & . & . & . & 0 & 3.9*f(\mathrm{Lleaf}) & 0 & . & . & . & . & 0 \\
0 & . & . & . & . & . & 0 & 4.8*f(\mathrm{Lfroot}) & 0 & . & . & . & 0 \\
0 & . & . & . & . & . & . & 0 & 3.6*f(\mathrm{Lwood}) & 0 & . & . & 0 \\
0 & . & . & . & . & . & . & . & 0 & 6.0 & 0 & . & 0 \\
0 & . & . & . & . & . & . & . & . & 0 & 7.3*f(\mathrm{clay\_silt}) & 0 & 0 \\
0 & . & . & . & . & . & . & . & . & . & 0 & 0.25 & 0 \\
0 & 0 & 0 & 0 & 0 & 0 & 0 & 0 & 0 & 0 & 0 & 0 & 0.0045
\end{pmatrix}
\begin{matrix}
leaf \\ stem \\ froot \\ croot \\ sml \\ fml \\ ssl \\ fsl \\ cd \\ sm \\ m \\ s \\ p
\end{matrix}
$$

$$
\begin{matrix} leaf & stem & froot & croot & sml & fml & ssl & fsl & cd & sm & m & s & p \end{matrix}
$$

The potential turnover rates are varied with biomes for vegetation pools. The values in surface metabolic, soil metabolic litter, surface microbe, slow and passive pools are directly defined with maximum turnover rates. Values in surface structure litter, soil structure litter and coarse detritus pools are defined by maximum turnover rates and factors from lignin contents in leaf (Lleaf), fine root (Lfroot) and stem(Lwood), respectively. Value in soil microbe pool is

defined by maximum turnover rate and soil texture factor (clay_silt).

Matrix ξ

ξ represents the environmental scalar to influence the decomposition rates in each carbon pools.

$$
\begin{array}{c}
\left(\begin{array}{ccccccccccccc}
1 & 0 & 0 & 0 & 0 & 0 & 0 & 0 & 0 & 0 & 0 & 0 & 0 \\
0 & 1 & & & & & & & & & & & 0 \\
0 & & 1 & & & & & & & & & & 0 \\
0 & & & 1 & & & & & & & & & 0 \\
0 & & & & \xi_T \times \xi_W & & & & & & & & 0 \\
0 & & & & & \xi_T \times \xi_W & & & & & & & 0 \\
0 & & & & & & \xi_T \times \xi_W & & & & & & 0 \\
0 & & & & & & & \xi_T \times \xi_W & & & & & 0 \\
0 & & & & & & & & \xi_T \times \xi_W & & & & 0 \\
0 & & & & & & & & & \xi_T \times \xi_W & & & 0 \\
0 & & & & & & & & & & \xi_T \times \xi_W & & 0 \\
0 & & & & & & & & & & & \xi_T \times \xi_W & 0 \\
0 & 0 & 0 & 0 & 0 & 0 & 0 & 0 & 0 & 0 & 0 & 0 & \xi_T \times \xi_W
\end{array}\right)
\begin{array}{c}
leaf \\ stem \\ froot \\ croot \\ sml \\ fml \\ ssl \\ fsl \\ cd \\ sm \\ m \\ s \\ p
\end{array}
\\
\begin{array}{ccccccccccccc}
leaf & stem & froot & croot & sml & fml & ssl & fsl & cd & sm & m & s & p
\end{array}
\end{array}
$$

In BEPS, environmental factors to decomposition in vegetation pools are not considered. For all the litter and soil pools, a combination of soil temperature (ξ_t) and soil moisture (ξ_w) is considered with same algorithms.

Text S2. Abbreviations annotation

Pool names:

Vegetation pools:

leaf: leaf pool; *stem*: stem(woody) pool; *froot*: fine root pool, *croot*:coarse root pool.

Litter pools:

sml: surface metabolic litter pool; *fml*: soil metabolic litter pool; *ssl:* surface structure litter pool; *fsl*:soil structure litter pool; *cd*: coarse detritus pool.

Soil pools:

sm: surface microbe pool; *m*: soil microbe pool; *s*: slow pool; *p*: passive pool.

Vegetation types:

EBF: evergreen broadleaf forest;
DBF: deciduous broadleaf forest;
ENF: evergreen needleleaf forest;
DNF: deciduous needleleaf forest;
MF: mixture forest;
GRA: grassland.

Table S1. Mean values and standard deviations of annual temperature and precipitation in various biomes.

Biomes	Temperature (℃)	Precipitation (mm)
EBF	24.1 (±3.7)	2140.3 (±683.4)
DBF	13.7 (±9.1)	1002.5 (±409.2)
ENF	0.51 (±4.4)	683.1 (±367.9)
DNF	-7.1 (±3.4)	394.1 (±149.4)
MF	6.8 (±13.1)	782.8 (±468.6)
Close Shrub	17.6 (±11.9)	816.4 (±509.9)
Grassland	7.6 (±12.7)	457.9 (±396.2)
Open Shrub	0.74 (±15.0)	325.7 (±193.4)
Wetland	8.7 (±13.0)	1119.3 (±816.1)
Crop	11.2 (±8.3)	699.4 (±414.2)
Crop/Nature	15.5 (±9.9)	1089.7 (±621.7)
Barren	18.0 (±10.8)	98 (±13.2)
Tundra	-17.5 (±6.6)	420.7(±318.2)

Table S2. Sensitivity of baseline residence time to its main determinants in various biomes. L/N: the ratio of lignin content to nitrogen, Lleaf: leaf lignin percentage, Lfroot: fine root lignin percentage, Lwood: woody lignin percentage, Soil Texture: Clay and Silt percentage in Soil.

Biomes	S value to baseline residence time				
	L/N	Lleaf	Lfroot	Lwood	Soil Texture
EBF	0.81	1.0	1.0	1.0	0.47
DBF	0.81	1.0	1.0	1.0	0.43
ENF	0.8	1.0	1.0	1.0	0.45
DNF	0.79	1.0	0.9	1.0	0.41
MF	0.83	1.1	1.0	1.1	0.51
Close Shrub	0.84	1.1	1.1	1.1	0.58
GRA	0.74	1.1	1.1	1.0	0.71
Open Shrub	0.84	1.0	1.1	1.1	0.58
Wetland	0.83	1.0	1.0	1.1	0.62
Crop	0.72	1.0	1.1	0.9	0.74
Crop/Nature	0.72	1.0	1.1	0.9	0.74
Barren	0.42	0.54	0.53	0.55	0.38
Tundra	0.83	1.0	1.0	1.0	0.62

Figure S1. Temperature scalar and moisture scalar differences between BEPS and CABLE scheme in various biomes.

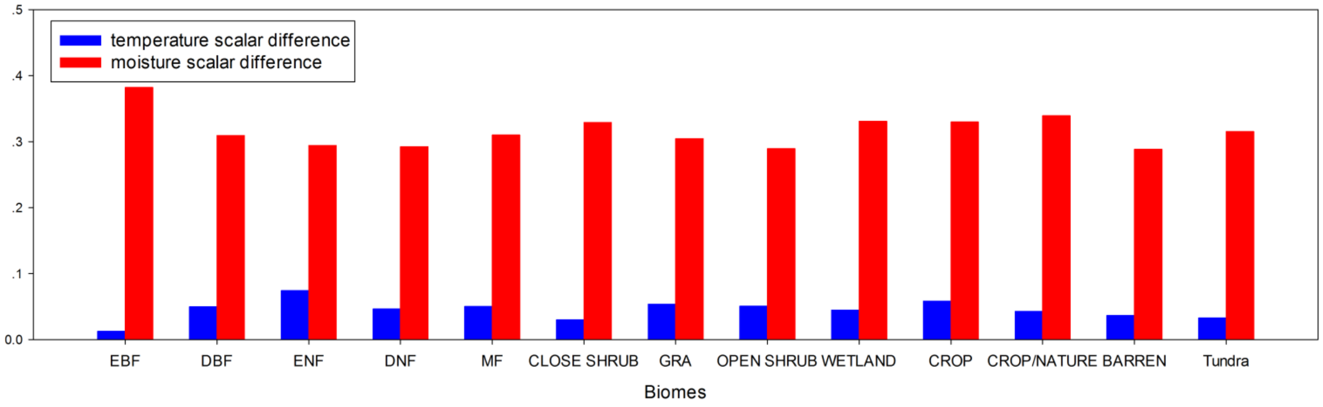

Figure S2. Global spatial and frequency distribution of moisture scalar differences by using different agents to represent soil water condition (soil water content versus soil water content modified by soil porosity) in BEPS model (The global spatial distribution component was created from authors' data using ArcGis 10.0 software (ESRI): www.esri.com/software/arcgis/)**.**

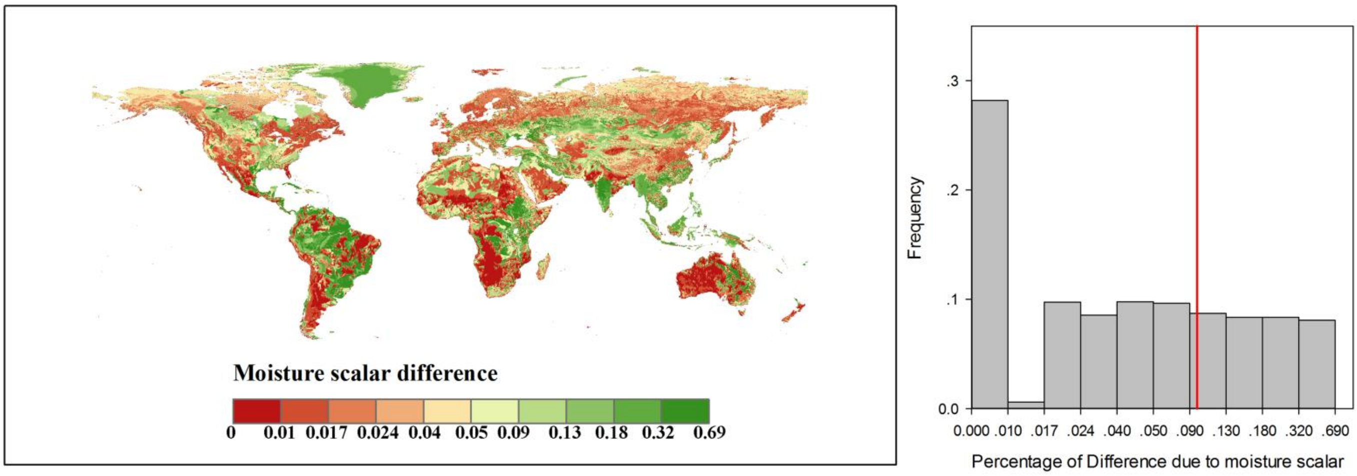

Figure S3. Global spatial and frequency distribution of moisture scalar differences by using different moisture scalar in BEPS (parabolic model with low limit) and ORCHIDEE (linear model) (The global spatial distribution component was created from authors' data using ArcGis 10.0 software (ESRI): www.esri.com/software/arcgis/)**.**

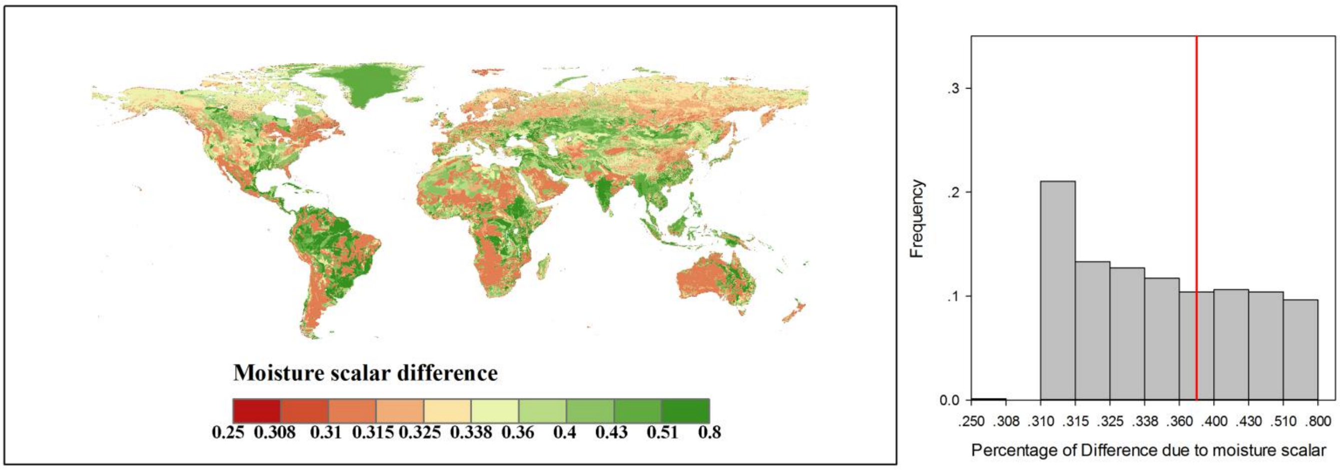

Figure S4. Moisture scalar differences between BEPS and CABLE scheme in various biomes. Yellow bars represent differences by using different agents to represent soil water condition in BEPS (BEPS-BEPS_nonpor, soil water content Versus soil water content modified by soil porosity); green bars represent differences by using the model in BEPS and CABLE (BEPS-CABLE); blue bars represent differences by using the linear model in ORCHIDEE and parabolic model in BEPS (BEPS-ORCHIDEE).

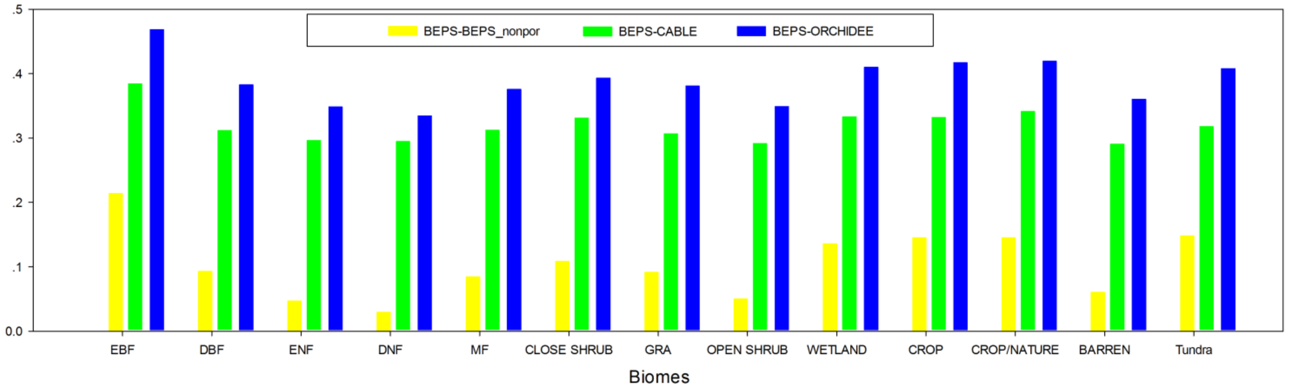

Supplement: Supplementary Information [file srep16155-s1.pdf]
